# Advances in Biosensors for Continuous Glucose Monitoring Towards Wearables

**DOI:** 10.3389/fbioe.2021.733810

**Published:** 2021-08-19

**Authors:** Lucy Johnston, Gonglei Wang, Kunhui Hu, Chungen Qian, Guozhen Liu

**Affiliations:** ^1^ School of Engineering, The University of Glasgow, Glasgow, United Kingdom; ^2^ School of Life and Health Sciences, The Chinese University of Hong Kong, Shenzhen, China; ^3^ Shenzhen YHLO Biotech Co., Ltd., Shenzhen, China; ^4^ The Key Laboratory for Biomedical Photonics of MOE at Wuhan National Laboratory for Optoelectronics, Hubei Bioinformatics and Molecular Imaging Key Laboratory, Systems Biology Theme, Department of Biomedical Engineering, College of Life Science and Technology, Huazhong University of Science and Technology, Wuhan, China

**Keywords:** diabetes, glucose biosensors, wearables, continuous monitoring, point-of-care detection

## Abstract

Continuous glucose monitors (CGMs) for the non-invasive monitoring of diabetes are constantly being developed and improved. Although there are multiple biosensing platforms for monitoring glucose available on the market, there is still a strong need to enhance their precision, repeatability, wearability, and accessibility to end-users. Biosensing technologies are being increasingly explored that use different bodily fluids such as sweat and tear fluid, etc., that can be calibrated to and therefore used to measure blood glucose concentrations accurately. To improve the wearability of these devices, exploring different fluids as testing mediums is essential and opens the door to various implants and wearables that in turn have the potential to be less inhibiting to the wearer. Recent developments have surfaced in the form of contact lenses or mouthguards for instance. Challenges still present themselves in the form of sensitivity, especially at very high or low glucose concentrations, which is critical for a diabetic person to monitor. This review summarises advances in wearable glucose biosensors over the past 5 years, comparing the different types as well as the fluid they use to detect glucose, including the CGMs currently available on the market. Perspectives on the development of wearables for glucose biosensing are discussed.

**GRAPHICAL ABSTRACT:**
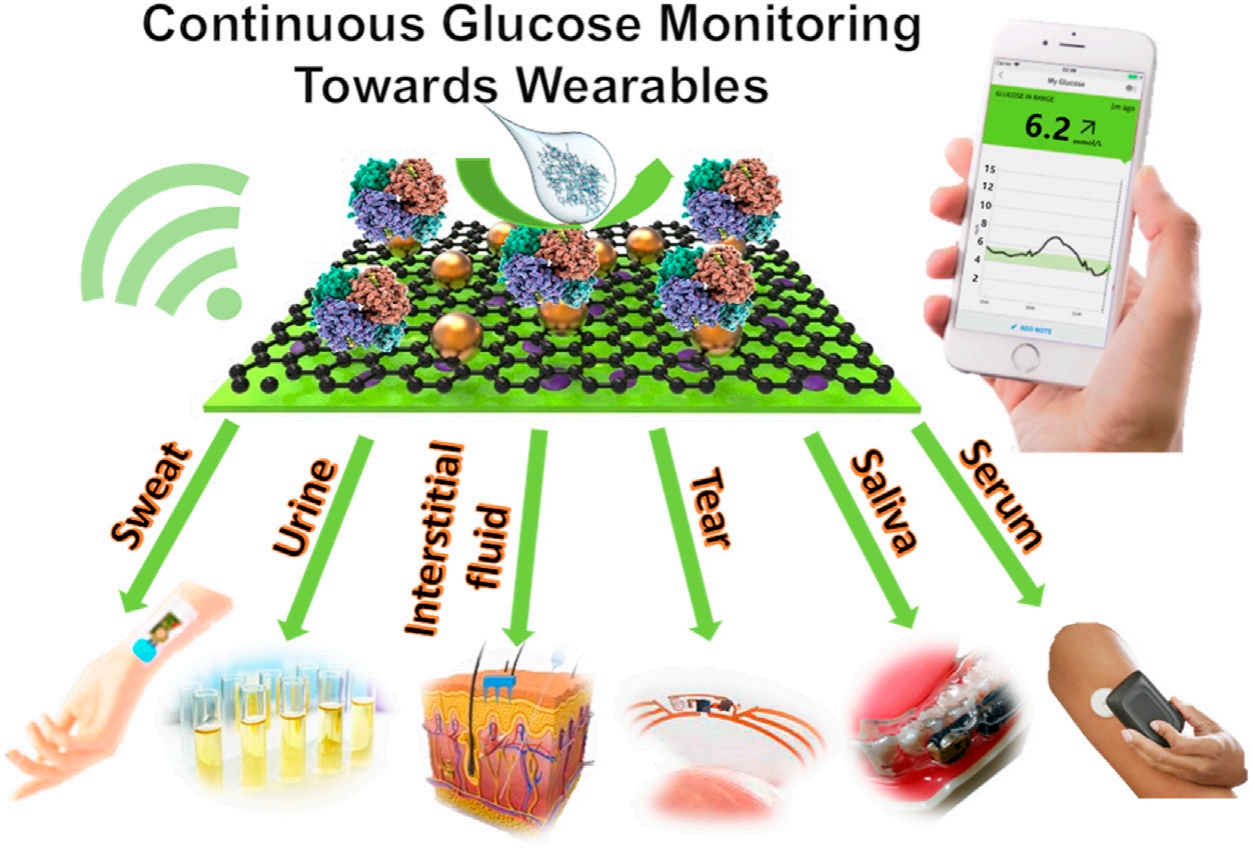


## Introduction

Diabetes is an intractable, challenging health problem in the 21st century. Worldwide, 422 million people have diabetes as of 2014, a lot of which reside in low- and middle-income countries, with the number of cases increasing steadily over the decades (WHO 2021). It is estimated to affect 552 million people globally by 2030 (Kharroubi and Darwish 2015). Diabetes is the major contributor to other chronic diseases, including cardiovascular disease and chronic kidney disease, and is the leading cause of blindness in adults, yet almost half of diabetes cases remain undiagnosed. As one of the leading causes of death in the world, with 1.6 million deaths per year, diabetes research has never been more important (Diabetes Australia 2020). To alleviate the deaths caused by diabetes, the WHO List of Priority Medical Devices for management of cardiovascular diseases and diabetes, released on June 30, 2021, will help policy-makers and healthcare providers prioritize the selection and procurement of medical devices for diabetes. This list contains hundreds of devices that are required for diabetes treatment and detection, from primary care facilities to highly specialized hospitals and devices needed for health emergencies such as hypo or hyperglycemic emergencies (WHO 2021). According to Diabetes Australia, 12% of global health expenditure is spent on diabetes, that is USD$673 billion (Diabetes Australia 2020). It is therefore important to focus on low cost and effective diagnostic tools and monitoring devices for diabetes.

There are three main types of diabetes–type 1, type 2, and gestational diabetes. Type 1 diabetes mellitus is a common chronic, metabolic disease characterized by high fasting glucose, in which beta cells from the islets of Langerhans in the pancreas can no longer produce insulin, needed to maintain blood glucose levels in the range of 4.0–5.5 mmol/L (Bruen et al., 2017). When the body fails to maintain this range, hypoglycaemia and hyperglycaemia occur, which if left untreated, lead to physiological complications and serious damage to major organs. In an undiagnosed person, symptoms of Type 1 diabetes include polyuria (excessive urination), polydipsia (thirst), constant hunger, weight loss and fatigue among others (WHO 2021). Type 1 diabetes cannot at present be prevented, but its monitoring is of upmost importance for people suffering from the disease. Type 2 diabetes is a result of the body’s lack of response to and/or production of insulin. This type of diabetes is the most prevalent in the world today, representing 85–90% of all cases (Kharroubi and Darwish 2015). As opposed to Type 1, this type is more readily managed with regular exercise, healthy eating and the control of blood pressure and lipids (WHO 2021), although insulin can still be required. Preventing people from developing type 2 diabetes will substantially reduce the risk of complications such as cardiovascular disease, blindness, and kidney failure. Strong international evidence shows that type 2 diabetes can be prevented by accurate management of the dysregulated blood glucose and insulin (Tuomilehto et al., 2001; Dunkley et al., 2014). Thus, real-time monitoring of glucose is essential for the prediction and prevention of diabetes and following interventions to manage diabetes. Finally, gestational diabetes occurs during pregnancy, affecting between 12 and 14% of pregnant women. However, the majority of gestational pregnancies are healthy pregnancies, producing healthy babies if a healthy eating and exercise plan is followed, and most women will no longer have diabetes after the baby is born (Kharroubi and Darwish 2015). Close monitoring of glucose levels is highly recommended by doctors, however, to guide healthy food and proper exercise for pregnant women.

The current most important and popular method for diabetes management is based on at home monitoring of blood glucose using the finger-pricking method, in which patients must try and maintain their daytime blood glucose levels between 4.4 and 6.7 mm/L, using a test strip and a glucose meter. However, this can become a burden, relying on a high frequency of testing to provide good glycaemic control (Benjamin 2002), and does not warn of hypoglycaemia occurrences. The wounds caused by needle sticks contain the risk of infection. For children patients, the pain may lead them to fear, avoid blood glucose detection and refuse medical treatment. Furthermore, this method can be painful and can lead to loss of feeling in the fingertips (Bruen et al., 2017). The finger-pricking blood test is known to provide unreliable data due to inaccurate blood sampling and can also cause infections due to the regular invasive tests. An alternative to this method is the non-invasive continuous glucose monitoring (CGM) method, which gives a comprehensive overview of a person’s glucose levels, as well as insulin needs (Bergenstal et al., 2021). A study done to compare the safety of CGM with and without routine blood glucose monitoring (RBGM) confirmed that it is as safe and effective to use CGMs as in RBGM in adults of type 1 diabetes at low risk of hypoglycaemia (Aleppo et al., 2017). In fact, when using a CGM, patients can significantly decrease their HbA_1c_ levels as opposed to usual care (Beck et al., 2017). In general, the use of CGM devices results in better glycaemic control and a lower frequency in insulin administration, although it seems more studies are needed to confirm this.

With the development of materials science, chemistry, engineering, computer science, and wireless technology, wearable technology has made a significant advance in the aspect of digital quantification of our daily health continuously. It normally requires a flexible substrate, a reliable sensor and an efficient signal report system. The current COVID-19 pandemic facilitates the market demand for tools for disease diagnosis, prevention and management. It also boosts the development of wearable sensing technology which is evidenced as an exciting and emerging field of the research field and is matching proudly forward from monitoring of physical exercise activity to health conditions (Kim et al., 2019; Garg, et al., 2021). Due to its non-invasive and point-of-care nature, wearables win their wide applications in healthcare service. Wearable devices offer a platform for continuous measuring and recording of physiological data in the form of a device that can be used for the tracking of a person’s current medical status, diagnosis, and treatment (Qiao, et al., 2020). A common wearable sensor is a smartwatch which tracks daily activities and body physical parameters such as heart rate, temperature, and sleep cycles. Wearables can also be used in the context of telehealth to monitor patients, predict diseases, and remind wearers to take medication or attend appointments by monitoring the chemical information such as oxygen levels, glucose and metal ions in our body fluids. Furthermore, wearables with dermal, oral, ocular, and cochlear interfaces are being developed to expand the methods for medical data collection (Yetisen et al., 2018). Although blood remains the most reliable medium for health monitoring, the focus of research has been on other body fluids such as saliva, tears and sweat. These mediums remove the need for the invasiveness of blood testing, which is often not preferred by patients, and introduce possibilities for new wearables that improve the quality of life of its users (Parlak et al., 2020).

This review summarises recent research activities on the development of different biosensors for continuous monitoring of glucose over the past 5 years. Specifically, the performance of different continuous glucose monitoring systems in the context of different bodily fluids is compared in terms of the sensitivity, linear range, and limit of detection (among other factors). Their applications and challenges in wearables are discussed, and future perspectives in wearable glucose biosensors are proposed. To our knowledge, this is the first comprehensive review on glucose biosensors in the direction of wearables adaptable to different body fluids.

## Wearables for Glucose Biosensing

With its capability to continuously monitor analytes, wearable sensing devices have provided attractive opportunities for providing information to assist in the prediction, diagnosis, and prevention of disease, enabling early treatment intervention (Qiao et al., 2020). As a non-invasive or minimally invasive physiological monitoring device, CGM is a successful example of wearable biosensors in the market for monitoring chemical components in matrix samples such as intertidal fluids. Wearable glucose monitors providing continuous blood glucose levels rely on the precision and accuracy of biosensors to relay blood glucose concentrations correctly and efficiently in real-time, 24 h per day. Biosensors consist of three parts: a recognition element called the bioreceptor, a signal transducer, and a signal displayer, usually an electronic system (Liu et al., 2019). The device detects the presence of a biological analyte such as a molecule or structure through its binding and relays the concentration of said analyte in the solution in question. There are many different classes of biosensors, varying binding type and output, the two main categories being catalytic biosensors, based on enzyme reactions, and affinity biosensors, relying on DNA and antibodies (Shukla et al., 2016). Many CGMs employ the enzymatic method, using the enzyme glucose oxidase (GOD) as the recognition molecule to bind with glucose. Enzyme glucose biosensors are specific and sensitive, but this method has its drawbacks, for instance its lack of stability, easily affected by temperature, pH changes and humidity. Thus, lots of efforts have been invested to study enzymatic biosensors to overcome these instabilities, such as using the artificial nanozyme (Vokhmyanina et al., 2020). Non-enzymatic biosensors show promising advantages, as they are not affected by factors puzzling enzyme biosensors (Lee et al., 2016). Aptamers can also be used as recognition molecules for the detection of glucose (Pan et al., 2020).

Glucose levels in sweat, saliva, urine, tears, and interstitial fluid have the potential to be correlated to blood glucose levels. The recent development of wearable biosensing devices that monitor glucose in these fluids shows promising results, and these CGMs are demonstrating their potentials as non-invasive, pain-free alternatives to the outdated and unreliable finger-pricking method to improve the quality of life of millions of people with diabetes (Benjamin 2002; Aleppo et al., 2017; Bruen et al., 2017). Glucose is detectable in different body fluids such as sweat, saliva, urine, tears and interstitial fluid. However, the requirements of designing wearables for monitoring glucose in different body fluids are different.

## Continuous Monitoring of Glucose in Different Body Fluids

Table 1 compares different properties (such as pH and viscosity) of different body fluids. The physiological concentration of glucose in different fluids is also listed in Table 1. It shows that interstitial fluids (2–22 mM) content comparable glucose concentration to that in blood (2–30 mM) which justifies currently most wearable glucoses biosensors are monitoring glucose in interstitial fluids. Urine, tears and sweat have similar concentration of glucose which is about 50 times less than that in blood. Comparing with glucose in other body fluids, glucose in saliva is the lowest (0.03–0.08 mM) for a healthy adult in fasting. In the following section, biosensors for continuous glucose monitoring in different body fluids are discussed in terms of their potentials for wearable glucose biosensing. Meanwhile, the performance of glucose biosensors in different body fluids is compared in Table 2.

**TABLE 1:** The physiological concentration of glucose in different fluids.

Body fluids	pH	Glucose concentration range^a^	Viscosity (Pa s)	References
Blood	7.35–7.45	2–30 mM	1.52–1.54	Corrie et al. (2015)
				(Heller and Feldman, 2008)
				(Gudmundsson and Bjelle, 1993)
Urine	4.5–8	0–0.8 mM	0.6–1.2	Corrie et al. (2015)
				(Lankelma et al., 2012)
				(Inman et al., 2013)
Interstitial fluid	7.2–7.4	2–22.2 mM	—	Corrie et al. (2015)
				(Koschinsky and Heinemann, 2001)
Saliva	6.2–7.6	0.03–0.08 mM	2–8	Baliga et al. (2013)
				(Ye et al., 2013)
				(Kazuya et al., 2016)
Tear	6.5–7.6	0.1–0.6 mM	1.5–3	Makaram et al. (2014)
				(Yao et al., 2011)
				(Tiffany, 1991)
Sweat	4.5–7	0.02–0.6 mM	0.92	Heikenfeld, (2016)
				(Moyer et al., 2012)
				(Corrie et al., 2015)

a Indicates the concentration of a healthy adult in fasting.

**TABLE 2:** A summary of body fluid-based continuous glucose monitoring (CGM) studies.

References	Fluid	Format	Recognition molecule	Signal readout	Sensitivity ${\text{uA mM}}^{-1}{\text{cm}}^{-2}$	Linear range	Detection limit	Assay time	Biosensor life and stability	Advantages
						mM	μM	sec	days	
Robinson et al., 2016	Urine	—	GOD	Electrochemistry	—	0–833	—	—	—	Albumin, urea and bilirubin can also be detected
Shitanda et al. (2019)	Urine	Diaper	GOD	Electrochemistry	—	1–25	—	—	—	Self powered
Sharma et al. (2016)	Interstitial fluid	Arm, dermis	GOD		—	0–30	500	15	—	Correlate with capillary bold glucometer
Lee et al. (2016)	Interstitial fluid	Skin, *epidermis*	Parylene, nanoporous Pt, and nafion	Electrochemistry	1,620	0–36	50	13	4 days	Stability, simplicity in fabrication, and reproducibility
Zong et al. (2017)	Interstitial fluid	Dermis	GOD	Electrochemistry	1,600	—	1	—	—	less invasive
Song et al. (2017)	Interstitial fluid	Neck	GOD	Electrochemistry	—	0.03–10	10	NA	15	Good selectivity, high SNR
Yoon et al. (2018)	Interstitial fluid	Subcutaneous tissue	nPt-GOD	Electrochemistry	68.4	0–30	30	<15	—	Wireless
Brown et al. (2018)	Interstitial fluid	Skin	Glucose-binding protein	Fluorescence	—	—	—	—	—	Skin irritations reduction
Bollella et al., 2019	Interstitial fluid	Skin	FAD-glucose dehydrogenase	Electrochemistry	405.2 ± 24.1	0.05–5	7	—	5 (12% lost)	—
									30 (18% lost)	
Malone-Povolny et al. (2019)	Interstitial fluid	Skin	GOD	Electrochemistry	382 ± 1.50	1–27	—	300	—	—
Liu et al. (2016)	Saliva	Mouth	GOD	Electrochemistry	67.93	0.05–1.5	3	—	90	Painless, disposable, low cost and good selectivity
Bollella et al. (2017)	Saliva	Mouth	GOD	Electrochemistry	3.1	0.02–30	6.2	5	20	Results correlate with blood glucose levels
Arakawa et al. (2020)	Saliva	Mouthguard	GOD	Electrochemistry	—	0.002–10	—	480	—	No pre-treatment needed, interference rejection membrane
Elsherif et al. (2018)	Tear	Lens	Phenylboronic acid	Electrochemistry	120	0–50	—	240	—	Home diagnostics
Kownacka et al. (2018)	Tear	Lower eyelid	—	Electrochemistry	—	0.1–1	—	—	—	Painless
Zou et al. (2019)	Tear	Lens	GOD	Electrochemistry	9.7	Max 12	9.5	—	30	Long service life
Sempionatto et al. (2019)	Tear	Eyeglass	GOD	Electrochemistry	—	—	—	—	—	No contact
Duong et al. (2020)	Tear	—	GOD	Optical	—	0.1–10	—	—	30	High sensitivity
Guler et al. (2017)	Serum	—	GOD	Electrochemistry	75.26	0.02–4.34	9	3	25	Cost efficient, promsing response
Wang et al. (2017)	Serum	—	GOD	Electrochemistry	0.773 NCR/decade	0.1–5	100	0.5	—	—
Song et al. (2017)	Serum	—	Glucose dehydrogenases (GDH)	Electrochemistry	25.1	0.025–17	8	—	—	Excellent electrical conductivity
Wang et al. (2018)	Serum	—	GOD	Optical	—	0.2–10	0.08	—	—	Optical technique
Cai et al. (2020)	Serum	Dorsal cervical areas	GOD	Electrochemistry	16.66	0–12	—	—	14	Low cost, simple
Bhide et al. (2018)	Sweat	Skin	GOD	Electrochemistry	—	0.047–0.305	5.5	—	0.17	Monitoring in ultra-low volume sweat
Xuan et al. (2018)	Sweat	Skin	GOD	Electrochemistry	82 -	0.1–2.3	5	12	8	Low cost
Yuan et al. (2019)	Sweat	Skin	GOD	Electrochemistry	—	0–5	0.1	—	14	—
Wiorek et al. (2020)	Sweat	Epidermis	Chitosan + GOD	Electrochemistry	−c	0.01–0.2	—	4–7	—	PH and temperature correction, more accuracy
Yoon et al., 2020	Sweat	Skin	Chitosan + GOD	Electrochemistry	4.622	Max 2.1	300	—	14	—
Guan et al., 2016	Buffer solution	—	C_e_O_2_/C_u_O nanocomposite	Electrochemistry	2.77	—	10	5–8	20	Biocompatible
Fei et al. (2016)	Buffer solution	Wrist	—	—	—	—	—	—	—	Self-cleaning
Soto et al., 2016	Buffer solution	—	GOD	Electrochemistry	3.82 ± 1.5	1–3	—	—	14	—
Cordeiro et al., 2018	Buffer solution	Brain	GOD	Electrochemistry	2.71	0–2,16	72.9	<1	—	Reliable monitoring the brain glucose changing
Zong et al., 2018	Buffer solution	Dermis	GOD	Electrochemistry	16	—	1	—	1.58	—
Mercer et al. (2019)	Buffer solution	—	GOD, GDH	Electrochemistry	—	—	—	—	—	Wireless, store data in the internet
Elsherif et al. (2019)	Buffer solution	—	3-(acrylamido)-phenylboronic acid, acrylamide	Optical	2.6	0–20	—	Fast	—	Low interference, high SNR
Chen et al. (2020)	Buffer solution	—	GOD	Electrochemistry	8.5	0.5–15	—	<2	60	Low interference

### Urine

Urine is a fluid naturally produced by the body, the analysis of which does not require forced extraction from the body, nor an implant to access it, such as in the case of blood. Thus, this fluid allows for the practical and non-invasive detection of glucose levels, that of which has been shown to correlate to blood glucose levels, by using the changing refractive index of urine with respect to glucose concentration (Robinson and Dhanlaksmi 2017), or the shift in resonance frequency of urine, which is proportional to glucose concentration (Koirala et al., 2018). A two-dimensional photonic crystal ring resonator-based sensor is presented as a device based on the variation of glucose concentration in urine which varies the refractive index of the urine, changing the output of the sensor (Robinson and Dhanlaksmi 2017). The sensor is tiny in size (11.4 × 11.4 µm) and predicts results without any delay, a very desirable feature in any biosensor in which the speed of results determines the health of the patient. The sensor can predict urea, albumin, bilirubin, and glucose concentration in the urine samples, as well as glucose concentration in blood due to its correlation to urine samples when normalized. The high performance of this sensor in glucose detection through urine shows its potential to be incorporated in medical applications.

**FIGURE 1:**
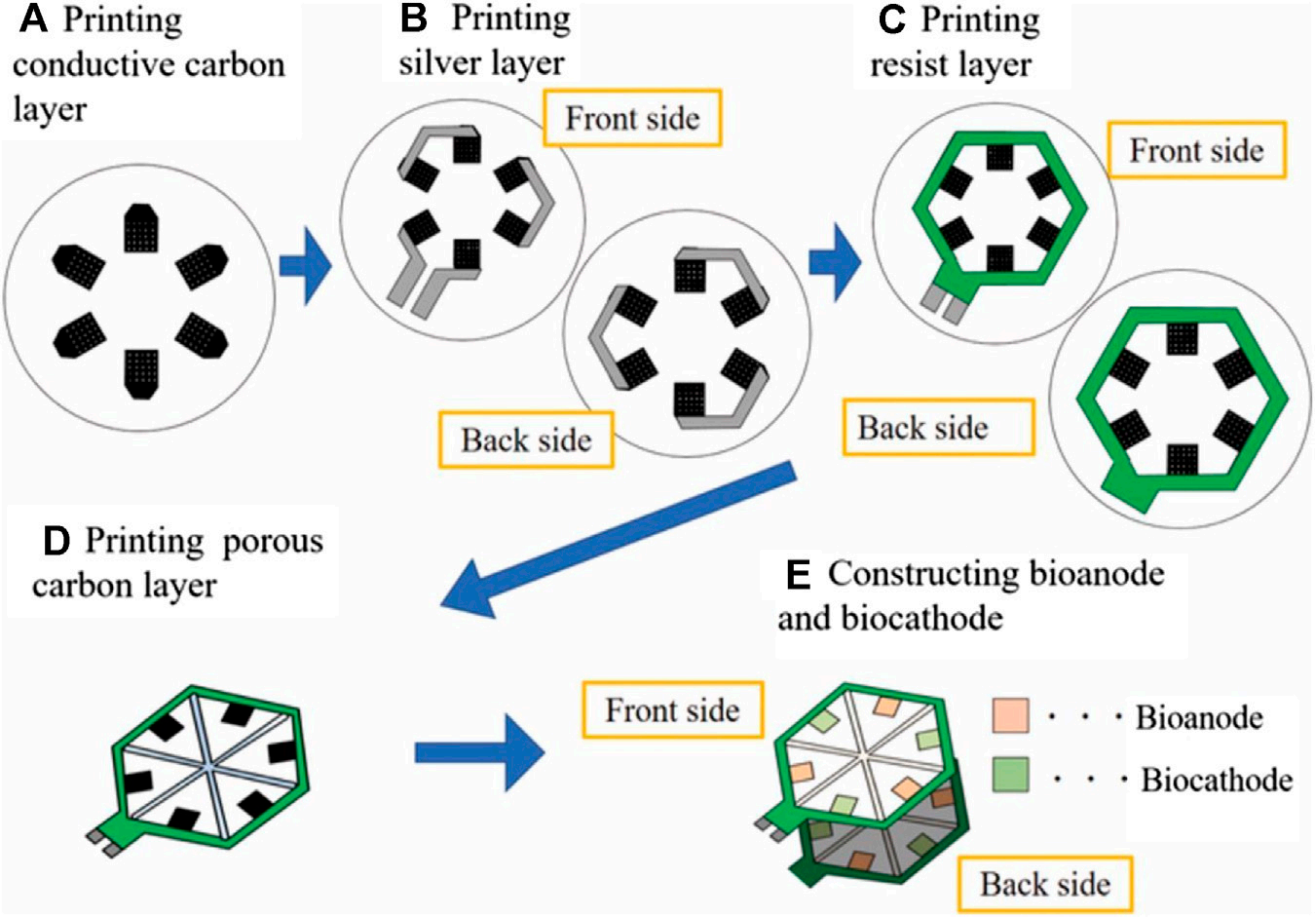
The process of the design and fabrication for the urine glucose test paper (Shitanda et al., 2019).

A paper-based disk based on screen-printed biofuel cell array is also proposed for the detection of glucose in urine with the electromagnetic signal readout by a resonator, for which the fabrication process is represented below in Figure 1 (Shitanda et al., 2019). A good correlation between the power output of the enzymatic biofuel cells and glucose concentration in the linear range of 1–25 mM was reported. Urine concentration is contained in this linear range, and it was reported that no components of the artificial urine tested interfered with the response of the biosensor. However, the device was limited by low ion conductivity and low buffer capacity. This self-powered glucose sensor, realised using enzymatic biofuel cells fabricated from screen printing, is incredibly useful in cases such as diabetics in nursing care and has possible future applications in diapers as wearables, but is not yet implicated as a continuous device.

These devices are expected to have many possible medical applications due to their ability to precisely detect glucose concentrations in a non-invasive way and could be implemented in future as implants or wearables. However, researchers are still striving to realize the actual wearable devices suitable for continuous urine glucose monitoring. The major challenges are associated with the matrix effect in urine on glucose (Kim et al., 2018), and also a reliable assay which is stable in urine (El-Farhan et al., 2017).

### Interstitial Fluid

**FIGURE 2:**
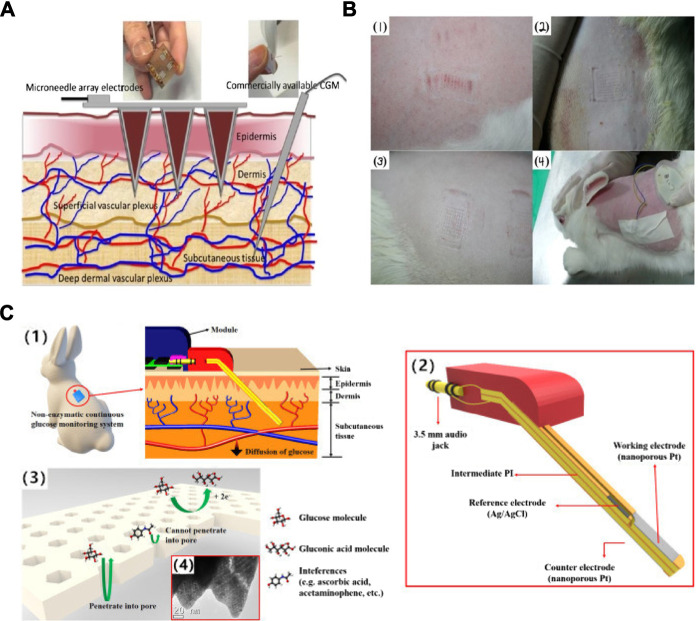
The schematic and profile map of the interstitial fluid-based CGMs. **(A)** Two methods (Microneedle array electrode && commercially CGM) detected the concentration of the blood glucose from the interstitial fluid (Sharma et al., 2016). **(B)** The photographs of insertion test of the sensor on (1) guinea pig, (2) rat, and (3) rabbit, and (4) the senor after being attached and partially inserted into a rabbit (Lee et al., 2016). **(C)** Semi-implantable device (1) a rodent worn the semi-implantable CGM. (2) the cross-section view of the device. (3), (4) Progress of oxidation reaction on nPt surface (glucose and interferences) and TEM graph (Yoon et al., 2018).

This first enzymatic biosensor aims to be minimally invasive and therefore inflict minimal pain, using metallic electrodes and microneedle technology as a detection method for glucose in interstitial fluid (IF) (Sharma et al., 2016). Figure 2A shows the penetration depth of the microneedles as oppose to commercially available CGM devices. Both access interstitial fluid, but the microneedles lie in the superficial vascular plexus as opposed to subcutaneous tissue, therefore they are less invasive and potentially reduce pain. The sensor showed good results both *in vitro*, with a detection limit of 0.5 mM and a response time of only 15 s, as well as *in vivo* in a healthy patient over a 3–6 h period. The results of this test revealed that the concentrations obtained by the biosensor can be correlated to capillary blood glucose concentrations in order to correctly predict blood glucose levels. Although the response of the device was lowered due to sterilization of the microneedles by gamma ray irradiation, performance was nevertheless adequate. This device is promising for the future, as it holds solutions to many problems that current devices have, such as painful application, accuracy, as well as cost. Another example also employed microneedles to obtain a minimally invasive device, however, this detection method is based on a non-enzymatic biosensor with amperometry readout (Lee et al., 2016). This wearable patch device was tested *in vitro* for 6 days before being tested on a rabbit for 4 days Figure 2B shows photographs of the insertion sites on (1) a guinea pig, (2) a rat, (3) a rabbit, and (4) the sensor attached to the rabbit. The results showed a high sensitivity of 1.62 μA mM^−1^cm^−2^ in a linear range of up to 36 mM, a fast response time of just 13 s, and a low detection limit of 50 µM. Although this device is promising and shows stability *in vitro*, the *in-vivo* test was halted after only 4 days of the planned six due to biofouling, displaying a shorter lifetime. Another device using a zinc oxide nanorod-based field effect transistor also aimed to be minimally invasive by reducing its sensing area to 180 μm^2 and an extremely low detection limit of 1 µM but at the price of a much lower sensitivity of 1.6 mA mM^−1^cm^−2^ (Zong et al., 2017). With a similar aim to reduce the pain and invasiveness that accompany current CGMs, all the while maintaining acceptable levels of sensitivity and reliability, a study used the microneedles to increase skin permeability and glucose across allowing for transdermal glucose to be used more effectively (Brown et al., 2018). A glucose-binding protein attached with a fluorescent probe was used as a measurement technique and due to its micromolar sensitivity, minimised the volume of fluid needed for an accurate prediction of glucose concentration. Overall, this device reduced application time and skin irritations but does not yet have continuous monitoring applications. This technology could be applied to CGMs in future to reduce invasiveness and pain caused to patients.

An open circuit potential biosensor was tested both *in vitro* and vivo using IF (Song et al., 2016). Glucose oxidase (GOD) loaded onto a microporous carbon derived from kenaf stem formed the electrode, which upon catalysis of glucose, changed the open circuit potential of the biosensor, without the need for an external energy supply. The results showed a linear range between 0.03 and 10 mM and a good detection limit of 10 µM. Although the linear range is not as wide as other sensors noted previously, the lower range of 0.03 is extremely useful to be measured especially during hypoglycaemias. Furthermore, the stability of this sensor is very reliable, retaining 96.9 and 95.8% of their original open circuit potential values after storage for 15 days at 4°C. The device also displayed good selectivity of glucose over common interferences and was tested *in vivo* in rat subcutaneous tissue with satisfactory results. The advantage of the open circuit potential method for glucose monitoring over classic methods such as spectrophotometry, colorimetry and electrochemiluminescence is that it is low cost, has high selectivity as well as easy operation and the ability to eliminate interfering molecules.

A semi-implantable device with a stainless-steel base and flexible electrochemical glucose sensor comprised of an electrode using nanoporous platinum coated with Nafion film shows a very good sensitivity of 684 nA mM^−1^ mm^−2^, a linear range of up to 30 mM, and a response time of 15 s (Yoon et al., 2018). This device makes use of hydrogen atoms on the platinum surface, which oxidise glucose molecules to glucono δ-lactone, using amperometry as a measurement method. Figure 2C(1) indicates the level of skin reached by the sensor as being subcutaneous tissue, Figure 2C(2) represents the three electrodes that make up the device. Finally, Figure 2C(3-4) shows how the pore size and shape are permeable to glucose molecules but reject interferences such as ascorbic acid. The material base of this sensor overcomes many biocompatibility changes, and *in-vivo* experiments conducted on rabbits show the implant to be promising for a future wearable sensor.

Additionally, an enzymatic, percutaneously implanted biosensor that measured nitric oxide (NO) release using amperometry for continuous glucose monitoring (Malone-Povolny et al., 2019). The NO release was tested using both porous and nonporous silica nanoparticles. This device was measured in a diabetic swine model over a 28 day period and showed that the release of NO correlates to glucose concentration and that the NO release technique is indicative of a lowered foreign body response caused by implantation. The sensitivity exhibited by the sensor was 3.82 ± 1.50 nA mM^−1^ over a 14 days release and revealed an acceptable linear range from 0 to 27 mM, but with a time response of 300 s. Currently, this sensor is limited by NO release duration but stays within a standard compliant accuracy for over 3 weeks. To increase the lifetime of this sensor beyond 1 month, research needs to be undertaken in this area in order to extend to NO release period.

Most of the devices that make use of IF as a measurement medium have a common goal to increase the sensitivity, lower sample volume, have a fast response time, and increase comfortability. Additionally, these wearables have very promising results, making use of microneedles, for example to reduce sensor size and increase skin permeability, or various new materials and sensing techniques to increase biocompatibility and overcome foreign body response issues.

### Saliva

The use of saliva as a medium for detecting glucose is first presented as a bienzyme and carbon nanotubes combined with a thin film (Liu et al., 2016). The detection mechanism uses a combination of GOD and horseradish peroxidase working in parallel with multi-walled carbon nanotubules by entrapping the enzymes in a chitosan matrix, whose role is to house the bienzymatic reactions. Polyphenol is employed as a semi-permeable membrane whose function is to reject any interferences that would otherwise hinder accurate glucose measurement and shows extremely good selectivity. The medium tested in this study is a phosphate buffer solution (PBS) with the addition of NaCl to simulate saliva, as well as *in vivo* on a test population of 30 individuals. Results revealed a sensitivity of 67.93 nA mM^−1^ as well as a linear range of 0.05–1.5 mM and a detection limit of 0.003 mM. Although the linear range is not very wide, the detection over the smaller concentrations is extremely precise, and the sensor’s ability to reject interfering constituents of saliva reflects a highly sensitive and selective biosensor. Furthermore, the biosensor has the capacity to be kept for up to 3 months at 4°C. This non-invasive, low cost and accurate sensor shows promising results for future applications but has yet to be improved for the detection of higher concentrations of blood glucose.

This next similar study aims to develop a biosensor that will correlate salivary glucose concentration to that of blood glucose concentrations for the development of a non-invasive glucose detection device (Bollella et al., 2017). This device uses a combination of nanotechnology to increase the electroactive area and third generation sensor technology to eliminate the mediator (a non-physiological electron acceptor) to develop a reagent-less biosensor that will transfer electrons from glucose directly to the electrode surface, resulting in a low operating potential. Glucose levels detected in saliva were compared with results obtained using standard spectrophotometry and blood glucose levels, showing excellent agreement in all cases. With a promising sensitivity of 3.1 ± 0.1 μA mM^−1^ cm^−2^, a detection limit of 6.2 μM, a very wide linear range of 0.02–30 mM and a fast response time of 5 s, this device could hold the potential to replace current glucose detection methods as a non-invasive, extremely reliable wearable technology.

**FIGURE 3:**
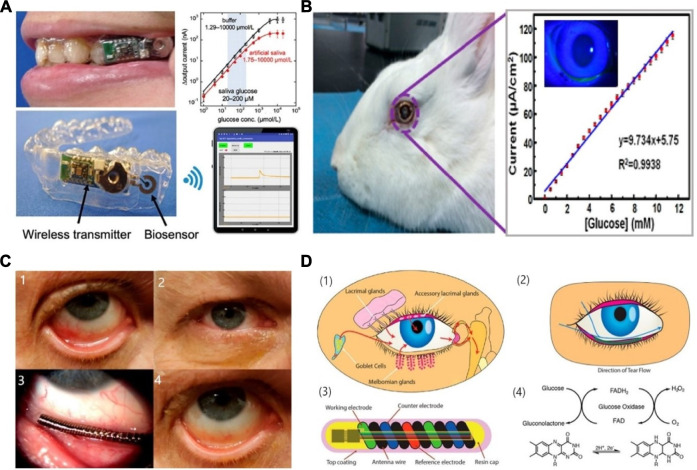
Saliva & tear fluid-based CGMs. **(A)** Mouthguard glucose sensor: structure, calibration curve and android app (Arakawa et al., 2020). **(B)** Eyeglasses glucose sensor was used in rabbit experiment and the calibration curve (Zou et al., 2019). **(C)** Eye implantable CGMs impacted on eyes. A: Baseline evaluation. The eye suffered mechanical rubbing and redness. B–D: Before/middle/after the trial, the eye did not show redness or damage (Kownacka et al., 2018). **(D)** Schematic illustration and properties of the CGMs. (1), (2) the tear fluid entered the device. (3) the structure of the tear glucose device. (4) Mechanism the glucose detection. Reaction for the GOD and glucose (Kownacka et al., 2018)*.*

This final biosensor applies the previously mentioned goal of a non-invasive device into a wearable biosensor in the form of a mouth guard (Arakawa et al., 2020). The mouthguard itself is coated with a cellulose acetate membrane whose function is to reflect any interferences found in the saliva, such as ascorbic acid (AA) and uric acid (UA). This wearable has many advantages, such as having high sensitivity and selectivity, a wide linear range of 1.75–10,000 μmol/L, as well as removing the need for pre-treatment of the saliva. In addition, an Android app was developed alongside the biosensor, and as shown in Figure 3A, which allows for wireless transmission of acquired data and portable monitoring. Although this wearable is able to measure low concentrations of glucose, valid for the detection of hypoglycaemias, it is not sufficiently sensitive at high concentrations, and therefore must be developed further to have a better linear range of detection.

Measuring glucose concentration in saliva needs to consider anti-interference capabilities due to the presence of salivary proteins and other inhibitory molecules, and this appears to be very well managed by certain biosensors (Liu et al., 2016; Arakawa et al., 2020), but a lack of sensitivity in the high glucose concentrations remain a common occurrence in biosensors and must therefore be developed further before being applied as a CGM (Iwai et al., 2016).

### Tear Fluid

Tear fluid, because of its easy accessibility and possible correlation to blood glucose, is becoming increasingly used as a fluid medium in wearable biosensors (Villiger et al., 2018; Choy et al., 2001; Zhang et al., 2011). March-investigated the glucose concentration between the blood and the tear and found these two values positively correlations (March, 2001). This first study focuses on the development of a biosensor that utilises the properties of an O_2_ sensing membrane and an immobilized layer of GOD on supporting materials to produce an enzymatic sensor displaying high sensitivity to low concentrations of glucose (Duong et al., 2020). The linear range of this device ranges from 0.1 to 10 mM, which is not as good as other sensors previously discussed, but the sensitivity in the low ranges of 0.1–2 mM is very high. The mechanism of glucose detection relies on fluoroscopy based on the amount of oxygen consumed during glucose oxidation. This device needs a lot of development towards measurements in the high glucose concentration ranges but is as a wearable or implantable device.

A wearable sensor, in the form of a coil in the shape of a spring that is placed in the inferior conjunctival fornix (under the lower eyelid) was developed (Kownacka et al., 2018). The polysaccharide hydrogel coating provides protection from any irritation or pain in the eye and provides a suitable surface on which GOD can be embedded. Figures 3C,D provides a graphical representation of the biosensor, including the enzymatic reaction occurring at the surface of the electrodes, as well as images of the sensor placed in the inferior conjunctival fornix.

The placement of the device allows for continuous monitoring by being bathed in the basal tear flow of the eye, and the flexibility of the coil allows it to take the shape of the wearers’ eye. The self-powered, wireless sensor exhibits the highest linearity in the range of 0.1–1 mM (Pandey et al., 2010; Liao et al., 2012; Blum et al., 2014). Scientists have explored four different approaches to power, including radiofrequency (RF) power (Cheng et al., 2013; Chiou et al., 2016), solar battery (Lingley et al., 2012), biofuel cell (Falk et al., 2012; Reid et al., 2015; Kulkami and Slaughter, 2017) and inductive power (Chen et al., 2017). *In vivo* testing revealed a good tolerance to interferences, as well as a performance similar to that of the FreeStyle Libre device available on the market. Current research on this device includes the ability of the sensor to detect fluctuations in interstitial blood glucose through tear fluid in relation to that of blood glucose. Although this device is non-invasive, it is perhaps uninviting to many people as a wearable because of the material and having to insert it under the eyelid might causing infection.

An alternative to this is the incorporation of a biosensor into contact lenses. This would be much more appealing to many users, and many people who are already using contact lenses for seeing purposes would perhaps find this innovative idea very enticing. This device utilises a contact lens to simplify the complex fabrication process involved in existing optical glucose sensors (Elsherif et al., 2018; Lin et al., 2018). As a result, a low-cost and reusable detector is developed that uses phenylboronic acid (PBA) as a recognition molecule. When binding to glucose, the volume of the hydrogel matrix in which the molecule resides is altered. When the volume changes, the periodicity of 3D photonic crystals that are also embedded in the hydrogel change, and this causes diffraction of specific wavelengths, which can be correlated to different glucose concentrations (Cameron and Anumula, 2006; Oliver et al., 2009). This impressive wearable displays a sensitivity of 12 nm mM^−1^, a linear range of 0–50 mM, and a response time of 3 s. The use of PBA in this device overcomes instability issues of other electrochemical sensors by using a photodetector and incident light technology instead. A smartphone readout was also developed that allows for at home monitoring of glucose concentrations. A similar intraocular biosensor has been researched, using nitrogen-doped graphene due to its high electroactivity, as well as carboxylated chitosan to exploit its high biocompatibility properties, interaction with enzymes and antibodies and hydrophilicity (Zou et al., 2019). This device shows a high sensitivity of 9.7 μA mM^−1^ cm^−2^, a significant linear range of up to 12 mM, and a detection limit of 9.5 μM. When worn by a New Zealand white rabbit for 24 h, no irritation nor unwanted side effects were reported. Figure 3B shows the intraocular lens being used by a white rabbit, as well as the current detected with respect to glucose concentration. This device maintains its parameters for up to 1 month in storage under 4°C, and does not use any toxic, flammable gases or high temperatures during production, and promises a great alternative to invasive and painful wearables. Contact lenses can be worn during the day, but during the night or when sleeping, users would have to revert back to other forms of glucose monitoring and would not be warned of hypoglycemic events.

Another study using tear fluid for glucose monitoring presents an innovative form of a wearable in the form of eyeglasses (Sempionatto et al., 2019). This device is used for the detection of alcohol and vitamins as well as glucose, but for the sake of this literature review, a focus will be made on its glucose sensing capabilities. The eyeglasses consist of wireless technology that sits outside the eye while collecting stimulated tears using an external miniaturized flow detector. This device is extremely interesting, as it eliminates all invasiveness, and would be very suited for people who already wear eyeglasses. However, the need for stimulation of tears, as opposed to the contact lenses or metal coil that passively collect tear fluid, seems inconvenient. It does, however, overcome issues connected with the contact lenses, such as infection and impaired vision. Further study is needed, to determine the device’s sensitivity and precision, to determine whether it could be used in future by diabetic patients.

### Serum

Serum, effectively blood plasma without the presence of fibrinogens, presents a useful medium for the detection of glucose, as will be shown. It is often accurate and reliable, although care must be taken due to the numerous proteins and biomarkers present in the fluid. A device based on functionalized reduced graphene oxide (rGO) uses PBS, then human blood serum samples to evaluate the effect of interfering molecules on the readings (Qi et al., 2016; Guler et al., 2017). A sensitivity of 75.26 μA mM^−1^ cm^−2^, a linear range of 0.02–4.340 mM and a detection limit of 9 μM shows a hopeful device for the detection of low concentrations of glucose using this device. It also provided reasonable selectivity as well as a steady state response of 95% reached in less than 3 s. However, the current response to glucose decreased by 2.7% after only 9 days. The sensor does show excellent electrocatalytic activity to glucose, however, and is very cost-effective. A second device also makes use of rGO technology, combined with polypyrrole nanowires, to construct fibre electrochemical transistors (Wang et al., 2017). Using rGO nanosheets increased and grew the number of nanowires, as well as enhanced the electroactivity of the transistors. The device showed great sensitivity, a linear range of 100 nM to 5 mM, as well as a speedy response time of 0.5 s in some cases. This device also shows favourable results towards detection of low concentrations of blood glucose, successfully tested *in vivo* in white rabbits, showing potential towards wearable applications.

A sensor was proposed based on a thin layer of mesoporous metal-organic frameworks covering carbon nanotubes (CNTs) surfaces as a platform on which to support electron mediators and the recognition molecule of glucose dehydrogenase (Song et al., 2017). The high surface area of the nanocomposites, as well as the great electroconductivity of the CNTs contributed to the development of a great biosensor with a large linear range of 25 μM to 17 mM, a detection limit of 8 μM and a sensitivity of 25.1 μA mM^−1^. *In vivo* tests carried out using human serum showed good selectivity when faced with interfering molecules, and the good reproducibility and the ease with which molecules immobilize onto the sensor’s surface gives the potential for development towards other biosensor types. Our next device employs a fluorescence quenching mechanism in the detection of glucose, using a combination of enzymatic reactions and graphene quantum dots (GQDs) to successfully create a linear relationship between glucose concentration in serum and the fluorescent intensity of the enzymatic reactions over a range of 0.2–10 μM, using enzymatic coupling of GOD and horseradish peroxidase (Wang et al., 2018). High sensitivity was achieved due to the optical properties of the GQDs, as well as high selectivity when faced with other molecules due to the enzyme coupling method.

**FIGURE 4:**
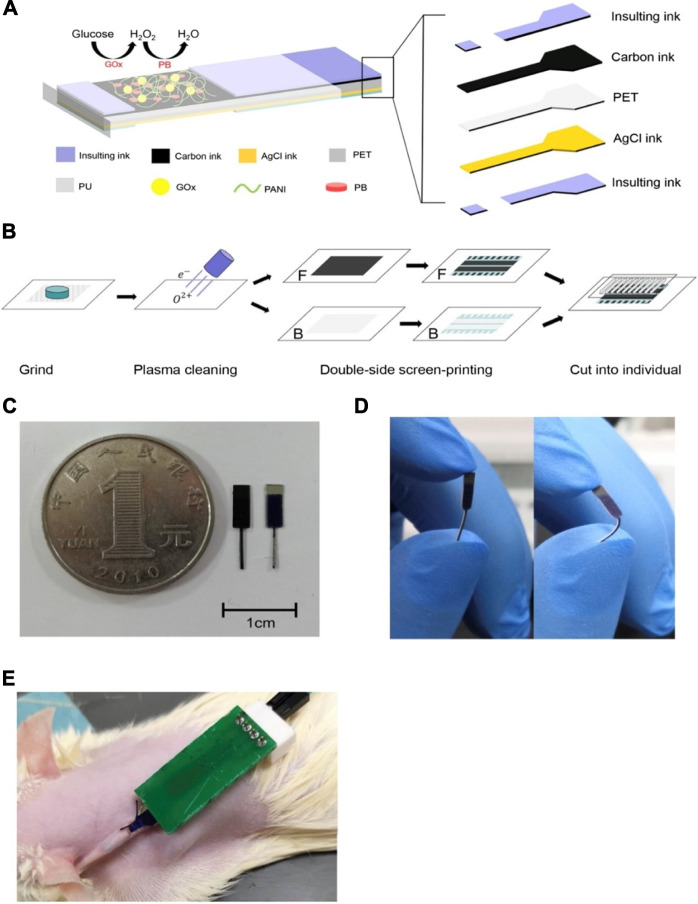
Serum-based CGMs (Cai et al., 2020). **(A)** The structure of the glucose sensor, including polyethylene terephthalate (PET) substrate, working electrode, a counter electrode and insulating material. **(B)** The array of the electrodes. **(C)** The shape of the electrodes. **(D)** Photograph of the electrodes binding at 90°. **(E)** The glucose monitoring tested on a rat.

Finally, a wearable application of a device utilizing the properties of serum as a testing medium for the detection of glucose is observed (Cai et al., 2020). This flexible biosensor is implanted in the dorsal cervical area and makes use of Prussian blue doped carbon ink that produces excellent specificity towards glucose by catalysing the reduction of hydrogen peroxide in low voltage conditions, thereby reducing interferences. The production of this sensor relied on a single-step modification of nano-polyaniline (PANI) and GOD, therefore making it simple, low cost, and applicable to large scale production. The fabrication process is outlined in Figure 4, which also illustrates the size and flexibility of the biosensor.

The application of the device in a white rat is shown in Figure 4E, which involves a small incision of 3–5 mm in the skin to allow implant under the skin. Results showed a linear range is of 0–12 mM which was enhanced using a polyurethane layer on the electrode surface, which also increased biocompatibility. The use of PANI increased the stability of GOD in the long term, useful for *in vivo* monitoring of blood glucose. Improvements in long term stability as well as sensitivity are still needed for this device, which is still very promising, nonetheless.

Although the use of serum as a medium for glucose detection is interesting, there is only one study that incorporates it into a wearable device, this being an invasive biosensor that would perhaps discourage its use when other non-invasive alternatives exist.

### Sweat

Sweat is an extremely useful medium for the measurement of glucose concentration in that it is passively produced by most humans. Our first study that utilises sweat for the detection of glucose is a single layer graphene-based sensor, coated in gold nanoparticles, themselves housing GOD, that exploit the properties of a glassy carbon electrode and 6-hexanethiol as an electron transfer medium (Yuan et al., 2019). The use of chemical vapor deposition for the gold nanoparticles simplified the production process of the sensor, creating a clean surface increased the immobilization capability of GOD, and the ductility monolayer graphene was made suitable for the detection of ultralow concentrations of glucose. Indeed, the linear range of the device was visible over 0.0005–5,000 μM and has a very low detection limit of 0.1 nM. The device did, however, lose 19% of its current response in just 2 weeks. This device is not yet wearable, but when tested in real human sweat, it shows great potential in the application towards perspiration-based glucose sensing. This next study takes a non-invasive wearable approach to perspiration-based glucose monitoring (Bhide et al., 2018). It makes use of ultra-low volumes of sweat for rapid and dynamic glucose monitoring, which can be applied to a wearable device in future. It takes only 2 h for measurements to stabilize, the same as most commercially available CGMs today.

**FIGURE 5:**
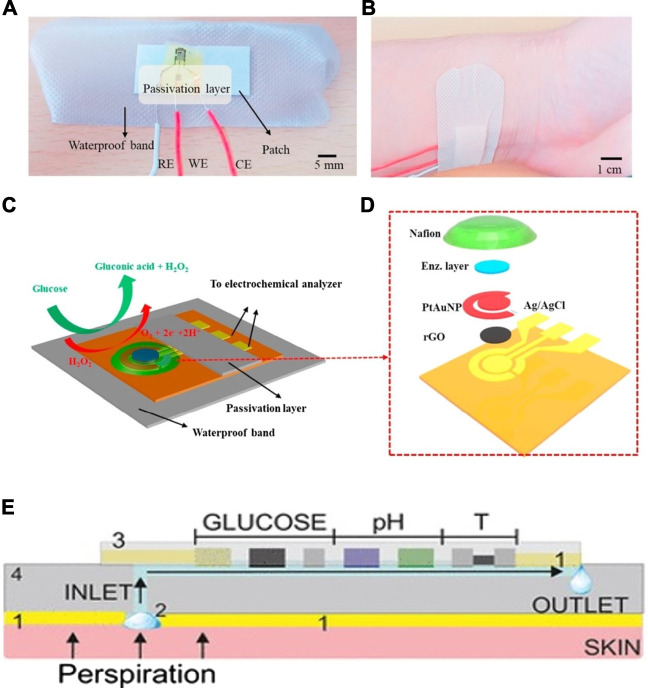
Schematics and mechanism of sweat-based CGMs. **(A,B)** Structure of the sensor and the sketch for the device applying on the skin (Xuan et al., 2018). **(C,D)** The reaction mechanism and the exploded view for the sensor (Xuan et al., 2018). **(E)** optimization of the device, minimum the temperature and PH impact on the result (Wiorek et al., 2020).

A wearable, low cost, electrochemical biosensor based on rGO film on which gold and platinum alloy nanoparticles were deposited is presented (Xuan et al., 2018). GOD and chitosan composites were then embedded onto the modified surface. The wearable patch and its electrode connections are shown in Figures 5A,B , as well as the enzymatic reactions (C) and the fabrication layers involved (D). This device is practical in terms of its reproducibility, as well as its flexible and disposable design for easy application, high sensitivity of 82 μA mM^−1^ cm^−2^ and high selectivity, and finally its ability to use very small volumes of perspiration for glucose analysis. Its response time (12 s) is slower than other devices, and it retains only 85% of its stability over just 8 days. However, future improvements for this device include incorporating pH, humidity and temperature sensing, parameters all linked to blood glucose concentration, to produce an even more sensitive device.

Another study includes pH and temperature corrections in its device for a more accurate analysis of sweat (Wiorek et al., 2020). Very similar to the previous research, this device uses the properties of GOD and chitosan composites, embedded on a wearable skin patch built as a microfluidic cell including a perspiration collection zone linked to a fluidic channel. This device is comprised of a glucose biosensor, a pH potentiometric electrode as well as a temperature sensor, illustrated in Figure 5E, all of which provide a comprehensive image of the dynamic fluctuations that occur in sweat. The response time is more rapid than the previous, at 4–7 s, but the linear range still resides in the lower glucose concentration range at 10–200 μM.

It seems that wearable biosensors based on the analysis of passive perspiration are extremely promising, especially in their accuracy at low glucose concentrations, and are relatively fast in response and stabilizing time, but lack the precision at higher glucose concentrations. This painless and innovative method of monitoring blood glucose needs more research to improve the range of measurement of blood glucose but is overall extremely hopeful in developing painless wearable biosensors.

## Commercially Available CGMs

Recently an excellent review has summarised the Continuous Glucose Monitoring (Funtanilla et al., 2019), in which 5 commercially available CGMs, FreeStyle Libre, Dexcom G4, Dexcom G5, Dexcom G6 and Medtronic Guardian Connect, were introduced. The review points out that the CGMs have the advantage over self-monitoring of blood glucose (SMBG) in that the devices measure blood glucose levels as often as every 5 min, meaning up to 288 times per day, giving a more comprehensive indication of a person’s trends and the ability to remain in the correct range for a larger portion of the day. Herein the performances and features of five commercial CGMs are compared in Table 3. It indicates that these CGMs are still relatively expensive, which might be a big hurdle for them to enter developing countries with a big population of prediabetes and diabetes. Additionally, sensor life is less than 2 weeks, which also leaves space for researchers to explore the improvement of sensor life. Table 3 also lists advances made in CGMs in recent years, such as the inclusion and development towards smartphone accessible data, an increased scanning distance, and greater freedom when it comes to the placement of the implant itself. One point to note is the slight decrease in the price of the Dexcom devices. Although the FreeStyle Libre device is cheaper, the Dexcom line is much more comprehensive in all fields and has the all-important audio alarms when the patient experiences a low or a high, which during sleep, for instance, can be lifesaving. Furthermore, most of the devices can be claimed with certain health insurance providers, but not all people have access to this, nor do they necessarily have access to the required funds for these devices that could offer a better quality of life for people with diabetes. It is, therefore, a priority to produce new, low-cost but effective and accurate devices that will be accessible to all.

**TABLE 3:** Comparative table of five Commercially available CGMs.

Sensor	FreeStyle libre	Dexcom G4 platinum with share	Dexcom G5 mobile	Dexcom G6	Medtronic guardian connect
Sensor Price/month	$129-$153	$349	$349	$349	$553
Sensor Life	14 days	7 days	7 days	7 days	—
Reader/Receiver Price	$84-$100 one time	$599/year	$599/year	$355/year	—
Transmitter Price	—	$599/6 months	$599/6 months	$475/6 months	$775/12 months
Reporting data	Trend graph, visual alerts	Trend graph, send data (IOS system)	Send data (IOS & Android system), Audio alarms	Send data, Audio alarms	Send data (IOS system)
Placement	Upper arm	Under the skin	Under the skin	Under the skin	—
Scanning Distance	1.5 inches	20 feet	20 feet	20 feet	—
Accuracy time and Calibration	no calibration needed	Calibration needed	Calibration needed	Calibration needed	Calibration needed
Age	18 +	2 years +	2 years +	2 years +	14–75 years old
Other	Remains accurate with acetaminophen use	—	—	Accurate with acetaminophen use. Reduce frequency of hypoglycaemias	Predict highs or lows 10–60 min beforehand

The recent Medtronic Guardian Connect shows promising developments, not only in its small number of components (sensor and transmitter only) but also its predictive technology that allows the user to learn more about environmental factors that affect them, as well as machine learning inclusion which allows for prediction of lows and highs a lot sooner than other devices that give real-time alerts. However, it must be noted that the high price of this new technology which means its access will be limited for most users, as well as its smaller age window for users. Thus, it is in high demand to develop wearables devices to continuously monitor the glucose levels in a non-invasive, cost-effective, and friendly to end-users fashion.

## Challenges and Perspectives

Diabetes is a chronic condition that occurs when the body either does not produce enough insulin or cannot effectively use the insulin it does produce. As one of the four major types of noncommunicable diseases (cardiovascular disease, diabetes, chronic respiratory diseases and cancer), diabetes continuous to cause a huge burden to the community globally. The WHO indicates that 85% of diabetics deaths occur in developing countries and a major reason is the lack of testing and monitoring equipment (WHO, 2021). Thus, lots of efforts have been invested to develop novel assays and device for diagnosis, treatment, management and prevention of diabetes. Detection of glucose in blood is still the golden standard method for diabetes diagnosis and management. In additional to blood, saliva, urine, sweat, interstitial fluids, and tears are also body fluids in which glucose is present in different levels, and glucose concentration in some fluids are coordinated with that in serum (Corrie et al., 2015). Currently, CGMs link with automated insulin delivery and thus accuracy of CGM devices is critical for delivering the right amount of insulin to patients (Tiffany, 1991).

With the development of nanotechnology, advanced materials and biomedical engineering, wearable sensing devices have been keeping on attracting wide attention in monitoring health conditions in real-time. In additional to the finger prick glucose monitoring by a portable glucose meter, wearable glucose meters provide a non-invasive tool for continuous glucose monitoring, and are currently one of the most successful wearable biosensors which are available in the healthcare market. There are several commercially available CGM products, however their high cost limits their wide accessibility to wide community of diabetes. Meanwhile, researchers are keeping on work hard to develop novel technologies for continuous glucose monitoring, aiming to increase the sensitivity, fast the assay time, and lower the sample volume, et al. However, there are several challenges to be considered in order to achieve a successful CGMs.

### Biofouling

CGMs usually need repetitive measurements during an extended period of time, so satisfactory accuracy and stability are critical to their admission by the market. Due to exposure to the biological liquid, the proteins, cells or macromolecules will accumulate on the biosensor surface and form biofouling (Jiang, et al., 2020). The rapid adsorption would interfere with the diffusion of the target analyte to the sensor surface, which leads to a slow signal response and reduces the biosensor accuracy. Current wearables measure glucose in complex body fluids such as interstitial fluids. False positive results could be generated due to the interferences. Two desired methods to address this problem are the excellent antifouling coating materials by the fabrication of a layer of biopolymers (such as poly ethylene glycol) or zwitterion molecules (Liu and Gooding, 2006), and the sensitive automatic calibration mechanisms. The sensor surface materials should be considered to minimize biofouling effects and exclude coexisting electroactive interference while retaining enzymes at the sensor surface and avoiding leakage of potentially toxic sensor components (Kim et al., 2018). Compared with the minimally invasive CGMs (interstitial fluid), non-invasive CGMs (urine, saliva, tear, sweat) would suffer from less biofouling because of the less biological solution contact.

### Sensor Lifetime

Table 2 shows the stability of CGMs. The shortest life span sensor is the interstitial fluid sensor (average 8 days). The saliva sensor has the longest life span (average 50 days). To our knowledge, currently the maximum sensor lifetime for a CGM is 14 days. End-users need to change a new glucose sensor after 14 days, which would increase the cost and inconvenient workload. Therefore, improving the bioreceptor stability and accuracy has a significant effect on the CGMs promotion and commercialisation. How to make the sensor to have longer shelf-life is essential. Using the artificial enzyme (such as nanozymes) as the glucose recognition molecules might be a feasible and reliable way to achieve a robust glucose sensor for wearables.

### Cost

As an attractive tool for point-of-care diagnostics, wearable sensors meet the ASSURED criteria (Liu, et al., 2021). They need to be affordable, sensitive, specific, user friendly, rapid and robust, equipment-free, and deliverable. Thus they are a particular class of products which are inexpensive, simple, and providing the real-time signal. As shown in Table 3, the price for the majority of CGMS is above $100. The high price of CGM is one of hurdles for end users who are in developing countries where large population of diabetes live. Development of cheap biocompatible materials for wearables such as using paper-based materials, and also simplification of the device design and recycling the transducers will help to decrease the cost.

### Calibration

Except for biofouling, other severe and uncontrolled environments should also be investigated, such as temperature, humidity and dust. These may affect the signal transmission of the receptors. Thus sensor calibration is essential. The glucose concentration in blood (2–30 mM) and other biofluids has a significant difference. Except interstitial fluid (2–22.2 mM), other body fluids such as saliva (0.03–0.08 mM), tear (0.1–0.6 mM), sweat (0.02–0.6 mM) are much lower than blood glucose. So large experiments to acquire a high accuracy and reliable calibration curve should be important. In future, there is a need for more intensive investigation to build anti-fouling, long life, low cost, calibration freeCGMs. There are lots of extensive ongoing research efforts and the huge diabetes market, the next few years several CGMs will be marketization and enter diabetics families.

In summary, a CGM having a long sensor life, being accurate and sensitive *in vivo*, being accessible to resource limited settings, and affordable to the community are always welcome in order to get close to physiological glucose homeostasis. It is also beneficial for diabetes early diagnosis, management and prevention.
